# Changes in clinical features of multiple system atrophy in Japan

**DOI:** 10.1016/j.prdoa.2020.100054

**Published:** 2020-04-22

**Authors:** Yusuke Tokuhara, Shohei Watanabe, Hiroo Yoshikawa

**Affiliations:** a Department of Internal Medicine, Division of Neurology, Hyogo College of Medicine, Nishinomiya 663-8501, Japan; b Department of Neurology, Nippon Life Hospital, Osaka 550-0006, Japan

**Keywords:** Multiple system atrophy, Phenotype, Epidemiological study, Age at onset, [^123^I]-meta-iodo benzylguanidine myocardial scintigraphy

## Abstract

**Introduction:**

Multiple system atrophy (MSA) is an adult-onset progressive neurodegenerative disease that causes parkinsonism, cerebellar ataxia, and/or autonomic failure. MSA is categorized as MSA with predominant cerebellar ataxia (MSA-C) or MSA with predominant parkinsonism (MSA-P) according to the cardinal symptom at diagnosis. MSA-C has been reported to be the predominant presentation in Japan to date. However, major epidemiological studies regarding MSA in Japan were carried out before 2006; thus, the recent advancement of various imaging studies remains unclear. This study aimed to investigate the clinical characteristics of the recent MSA patients in Japan.

**Methods:**

In this retrospective study, we divided 80 probable MSA patients into group A and group B and examined them to reveal whether the clinical features of MSA were different depending on the time periods of diagnosis (1989–2003 and 2004–2018, respectively).

**Results:**

The age at onset was significantly higher in MSA-P patients than in MSA-C patients (*p* = 0.0039) and was also higher in group B than in group A (*p* = 0.013). Although MSA-C was the predominant type in both groups, MSA-P was significantly more frequent in group B than in group A (*p* = 0.039). Although not statistically significant, the early heart/mediastinum ratio in [^123^I]-meta-iodo benzylguanidine (MIBG) myocardial scintigraphy tended to be lower in patients with MSA-P than in those with MSA-C.

**Conclusion:**

The proportion of MSA-P was likely larger than previously recorded due to the aging population in Japan and the improvement of differential diagnosis between PD and MSA-P.

## Highlights

• We investigated the clinical features of 80 probable MSA patients.
• We compared two groups of probable MSA patients according to the period of diagnosis.
• MSA with predominant cerebellar ataxia (MSA-C) was predominant in both groups.
• MSA with predominant parkinsonism (MSA-P) increased in the recent diagnosis group.
• Aging and development of examination tools may have influenced these results.

## 1 Introduction

Multiple system atrophy (MSA) is an adult-onset progressive neurodegenerative disease that causes parkinsonism, cerebellar ataxia, and/or autonomic failure (e.g., orthostatic hypotension, urinary incontinence, and erectile dysfunction and so) [1]. There are two phenotypes of MSA, MSA with predominant cerebellar ataxia (MSA-C) and MSA with predominant parkinsonism (MSA-P). Formerly, these phenotypes were considered as different diseases and were called olivopontocerebellar atrophy (OPCA) and striatonigral degeneration (SND), respectively. Since the discovery of the presence of glial cytoplasmic inclusions (GCI) containing α-synuclein in the oligodendrocytes in both OPCA and SND, they have been regarded as the same disease - MSA [2]. The first consensus criteria for the diagnosis of MSA were established in 1999. These criteria focused on the clinical features, mainly parkinsonism, cerebellar dysfunction, and autonomic failure at diagnosis [3]. The diagnostic criteria classified the diagnostic certainty into either probable- or possible-MSA and decided that cases with GCIs were definite-MSA. The second consensus criteria for the diagnosis of MSA was established in 2008; various imaging findings and red-flag signs were newly adopted as additional features [4]. Clinical differential diagnosis between MSA and other neurodegenerative parkinsonism is often difficult (e.g., Parkinson's disease (PD), dementia with Lewy bodies, and progressive supranuclear palsy and so). Recently, various imaging studies for the differential diagnosis of Parkinsonism have developed. For example, [^123^I]-meta-iodo benzylguanidine (MIBG) myocardial scintigraphy is useful for differentiating PD from other syndromes with parkinsonism [[5], [6], [7]].

According to previous reports, MSA-P is more predominant than MSA-C in the United States and the European Union [8,9], while MSA-C is more frequent (67–84%) than MSA-P (16–33%) in Japan [10,11]. However, these large-scale epidemiological studies regarding Japanese MSA patients were published before 2006, and the recent advancement of various imaging studies remains unclear. Therefore, it is necessary to perform a new epidemiological study regarding Japanese MSA patients.

This study aimed to reveal whether the clinical characteristics of the recent MSA patients in Japan were different depending on the time periods of diagnosis.

## 2 Methods

### 2.1 Selection of patients and clinical evaluation

We retrospectively examined the medical records of 113 patients who were clinically diagnosed with probable or possible MSA at the Hyogo College of Medicine Hospital between 1989 and 2018. Although these patients were described as having either OPCA/MSA-C, SND/MSA-P, or Shy-Drager syndrome in the medical records, we reevaluated their clinical phenotype according to the second consensus criteria [4]. Firstly, we excluded 13 patients who did not meet the criteria for either probable or possible MSA. Secondly, we excluded 17 possible MSA patients. All of these possible MSA patients did not have sufficient autonomic failure to fulfill the second consensus criteria. Then, we also excluded 3 probable MSA patients whose medical information was insufficient to allow further investigation. As a result, we investigated 80 probable MSA patients. As we could not perform autopsies on any patients, we were unable to diagnose definitive MSA. Next, we separated the probable MSA patients diagnosed between 1989 and 2003 into group A, and the patients diagnosed between 2004 and 2018 into group B. We then assessed the groups to determine the gender ratio, clinical phenotypes, the age at onset, the time from onset to diagnosis, initial symptoms, clinical features at the latest follow-up including activity of daily living (bedridden, wheelchair-bound, aid-requiring walking or independent gait), autonomic failure (neurogenic bladder, orthostatic hypotension and impotence), and imaging findings.

The ethics committee of the Hyogo College of Medicine approved this study (approval number: 2941).

### 2.2 Imaging examinations

In this study, brain magnetic resonance imaging (MRI) was performed in 78 probable MSA patients to acquire T2 weighted axial images. Among them, 25 patients were examined with a 3.0 T scanner, 11 patients with a 1.5 T scanner and 6 patients with a 0.5 T scanner [3000–4700 ms repetition time (TR), 80–126 ms echo time (TE), 1–1.5 mm interslice gap, 4–7 mm slice thickness]. Unfortunately, we could not obtain enough information about the conditions of how it was performed in the remaining 36 patients (in particular for group A). Therefore, we did not compare MRI findings between groups. We evaluated the presence of pontine abnormalities (hot cross bun sign or atrophy), putaminal abnormalities (hyperintense putaminal rim sign or atrophy), and cerebellar atrophy, according to descriptions in the medical records by neurologists or radiologists.

[^123^I]-MIBG myocardial scintigraphy was performed in 21 probable MSA patients diagnosed between 2006 and 2018. Data were collected for 15 and 180 min after injection of 111 MBq [^123^I]-MIBG (FUJIFILM Toyama Chemical Co., Ltd., Tokyo, Japan) using a gamma camera FORTE (ADAC Laboratories, USA) and the data processing software PEGSYS (ADAC Laboratories, USA). A static image was acquired with a 256 × 256 matrix. Regions of interest (ROI) were manually drawn around the heart and mediastinum, and the [^123^I]-MIBG uptake in each ROI was measured to calculate the heart/mediastinum (H/M) ratio. As we could not obtain all the raw data, we did not conduct standardization of each value.

The cut off value for the H/M ratio was set to 2.0 according to the standard value used in the Hyogo College of Medicine Hospital. We compared early and delayed H/M ratios between MSA-C and MSA-P patients. Furthermore, to compare the H/M ratio between MSA patients and PD patients, we assessed 24 PD patients who were diagnosed with the International Parkinson and Movement Disorder Society clinical diagnostic criteria and underwent MIBG myocardial scintigraphy between May 2016 and March 2018.

Dopamine transporter single-photon emission computed tomography (DAT SPECT) was performed in 8 probable MSA patients (2 MSA-C patients and 6 MSA-P patients). Data were collected for 180 min after injection of 167 MBq [^123^I] ioflupane (DaTSCAN, Nihon Medi-Physics, Tokyo, Japan) using a gamma camera Bright View X with CT (Phillips, Eindhoven, Netherlands) and the image processing software EBM-NM (Philips, Eindhoven, Netherlands). The matrix size was 128 × 128 and the pixel size was 3.3 mm. Specific binding ratios (SBRs) were calculated using DaTView software (Nihon Medi-Physics, Tokyo, Japan). The asymmetry index (AI) of SBRs was calculated using the following equation: AI [%] = (SBR predominantly affected − SBR less affected × 2/(SBR predominantly affected + SBR less affected) × 100 [12]. To compare SBRs and AIs in DAT SPECT between MSA patients and PD patients, we assessed 17 PD patients who were diagnosed with the International Parkinson and Movement Disorder Society clinical diagnostic criteria and underwent DAT SPECT between October 2017 and March 2018.

### 2.3 Statistical analysis

The threshold for significance was set at *p* < 0.05. We performed the Mann-Whitney *U* test or Kruskal-Wallis test and the method of Holm to compare continuous variables, and performed Fisher's exact test to analyze cross-tabulation. All statistical analyses were performed with EZR version 1.38 (Saitama Medical Center, Jichi Medical University, Saitama, Japan), which is a graphical user interface for R (The R Foundation for Statistical Computing, Vienna, Austria) [13].

## 3 Results

### 3.1 Patient profiles

**Fig. 1:**
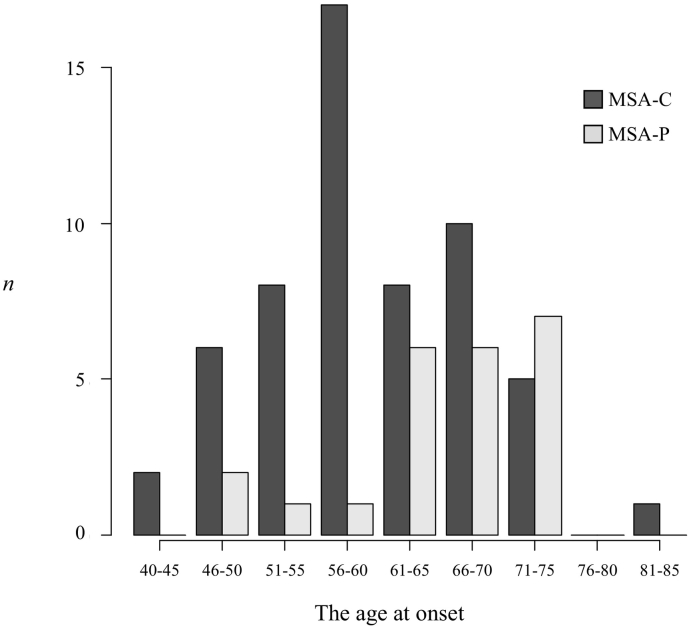
Age distribution of MSA-C and MSA-P. MSA-C occurred most often in the ages of 56 and 60 years, while MSA-P occurred more often in patients 61 or over years of age. MSA-P was predominant only in patients who were 71 to 75 years of age at syndrome onset compared to patients diagnosed with MSA-C.

The patients who met the criteria for probable MSA consisted of 42 men and 38 women (Table 1). Of these 80 patients, 57 patients were diagnosed with MSA-C (71%) and 23 patients were diagnosed with MSA-P (29%). The mean age at onset was 61.5 ± 8.5 years in all the MSA patients, 60.0 ± 8.5 years in the MSA-C patients, and 65.3 ± 7.4 years in the MSA-P patients. The mean age at onset was significantly higher in those diagnosed with MSA-P than in those diagnosed with MSA-C (*p* = 0.0039). MSA-C occurred most often in patients who were between 56 and 60 years of age, whereas MSA-P occurred more often in patients over 61 years of age. MSA-P was predominant only in patients who were between 71 and 75 years of age (Fig. 1). The mean time from onset to diagnosis was 32.3 ± 20.9 months in all patients, 30.4 ± 20.5 months in the MSA-C patients and 36.7 ± 21.5 months in the MSA-P patients. There was no significant difference in the time from onset to diagnosis between patients diagnosed with MSA-P and MSA-C. Initial symptoms included motor symptoms (parkinsonism and/or cerebellar ataxia) and autonomic symptoms (neurogenic bladder and/or orthostatic hypotension decreasing by 30 mmHg systolic or 15 mmHg diastolic). Motor symptoms were predominant in both MSA-C and MSA-P. There was no significant difference in the proportion of initial symptoms between patients diagnosed with MSA-C and MSA-P. The mean follow-up periods were 53.7 ± 39.2 months in all patients, 55.2 ± 42.5 months in the MSA-C patients and 49.9 ± 29.8 months in the MSA-P patients. There was no significant difference in the follow-up period. The patients in a bedridden state were predominant in both MSA-C and MSA-P (33% and 39%, respectively). There was no significant difference in ADL between patients with MSA-P and MSA-C.

**Table 1:** Profiles and clinical features of MSA patients.

	Total MSA			Group A 1989–2003			Group B 2004–2018		
	MSA	MSA-C	MSA-P	MSA	MSA-C	MSA-P	MSA	MSA-C	MSA-P
Number of patients (%)	80	57 (71)	23 (29)	29	25 (86)	4 (14) a	51	32 (63)	19 (37) a
Male/female	42/38	31/26	11/12	15/14	12/13	2/2	27/24	18/14	9/10
The age at onset	61.5 ± 8.5	60 ± 8.5 b	65.3 ± 7.4 b	58.4 ± 7.3 c	57.8 ± 6.9	62.3 ± 10.7	63.3 ± 8.5 c	61.7 ± 9.2	65.9 ± 6.8
The time from onset to diagnosis (month)	32.3 ± 20.9	30.4 ± 20.5	36.7 ± 21.5	32.2 ± 19.1	32.9 ± 20.3	28 ± 8.7	32.3 ± 22	28.5 ± 20.8	38.6 ± 23.1
Initial symptoms									
Motor (%)	58 (73)	39 (68)	19 (83)	20 (69)	18 (72)	2 (50)	38 (75)	21 (66)	17 (89)
Autonomic (%)	25 (31)	20 (35)	5 (22)	9 (31)	7 (28)	2 (50)	16 (31)	13 (41)	3 (16)
At the latest follow-up									
Follow-up period (month)	53.7 ± 39.2	55.2 ± 42.5	49.9 ± 29.8	52.3 ± 38.3	55.7 ± 39.0	23.3 ± 12.7	54.5 ± 40.1	54.7 ± 45.8	54.1 ± 29.7
Activities of daily living (ADL)									
Bedridden (%)	28 (35)	19 (33)	9 (39)	9 (31)	8 (32)	1 (25)	19 (37)	11 (34)	8 (42)
Wheelchair (%)	20 (25)	14 (25)	6 (26)	6 (21)	5 (20)	1 (25)	14 (27)	9 (28)	5 (26)
Aid-requiring walking (%)	16 (20)	9 (16)	7 (30)	6 (21)	4 (16)	2 (50)	10 (20)	5 (16)	5 (26)
Independent gait (%)	16 (20)	15 (26)	1 (4)	8 (28)	8 (32)	0	8 (16)	7 (22)	1 (5)
Neurogenic bladder (%)	69 (86)	48 (84)	21 (91)	26 (90)	22 (88)	4 (100)	43 (84)	26 (81)	17 (89)
Urinary incontinence (%)	41 (51)	30 (53)	11 (48)	15 (52)	14 (56)	1 (25)	26 (51)	16 (50)	10 (53)
Frequent urination (%)	11 (14)	8 (14)	3 (13)	4 (14)	3 (12)	1 (25)	7 (14)	5 (16)	2 (11)
Dysuria (%)	17 (21)	10 (18)	7 (30)	7 (24)	5 (20)	2 (50)	10 (20)	5 (16)	5 (26)
Orthostatic hypotension (%)	58 (73)	42 (74)	16 (70)	21 (72)	18 (72)	3 (75)	37 (73)	24 (75)	13 (68)
>30 mmHg systolic or 15mHg diastolic (%)	50 (63)	36 (63)	14 (61)	18 (62)	15 (60)	3 (75)	32 (63)	21 (66)	11 (58)
>20 mmHg systolic or 10mHg diastolic (%)	8 (10)	6 (11)	2 (9)	3 (10)	3 (12)	0	5 (10)	3 (9)	2 (10)
Impotence (%)	6 (14)	6 (19)	0	2 (13)	2 (17)	0	4 (15)	4 (22)	0

MSA, multiple system atrophy; MSA-C, multiple system atrophy with predominant cerebellar ataxia; MSA-P, multiple system atrophy with predominant parkinsonism. Group A consist of patients diagnosed as MSA between 1989 and 2003, Group B consist of patients diagnosed as MSA between 2004 and 2018.

a *p* = 0.039 by Fisher's exact tests.

b *p* = 0.0039 by Mann-Whitney *U* test.

c *p* = 0.013 by Mann-Whitney *U* test.

Regarding the autonomic symptoms (neurogenic bladder, orthostatic hypotension and impotence), neurogenic bladder was the predominant symptom in both MSA-C and MSA-P (84% and 91%, respectively). Among them, urinary incontinence was predominant in both groups (53% and 48%, respectively). There was no significant difference in the occurrence rate of each autonomic symptom between the patients diagnosed with MSA-C and MSA-P.

**Fig. 2:**
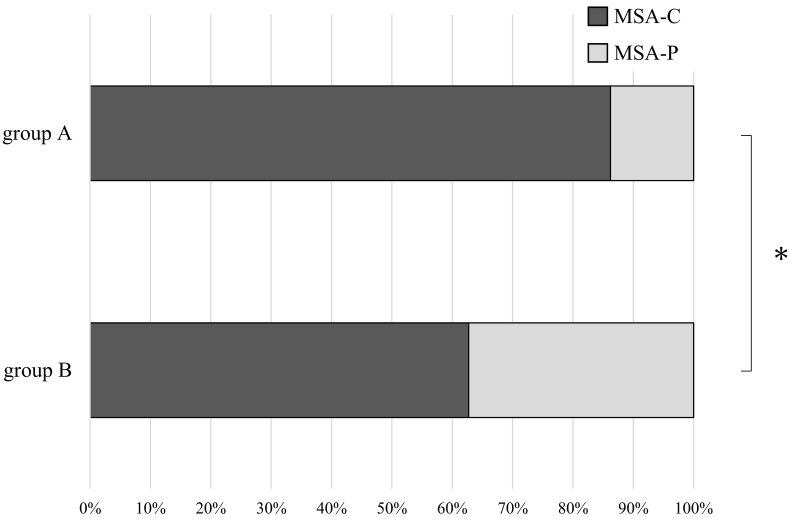
Comparison of the proportion of MSA-C and MSA-P between the groups. The proportion of MSA-P was significantly higher in group B than in group A.

There were 29 patients in group A and 51 patients in group B (Table 1). Group A consisted of 15 men and 14 women, and group B consisted of 27 men and 24 women. There was no significant difference in the gender ratio between groups A and B. The mean age at onset was 58.4 ± 7.3 years in group A patients and 63.3 ± 8.5 years in group B patients. The mean age at onset was significantly higher in group B than in group A (*p* = 0.013). In group A, 25 patients were diagnosed with MSA-C (86%) and 4 patients were diagnosed with MSA-P (14%). In contrast, in group B, 32 patients were diagnosed as MSA-C (63%) and 19 patients were diagnosed as MSA-P (37%). The proportion of MSA-P was significantly higher in group B than in group A (*p* = 0.039) (Fig. 2). The mean time from onset to diagnosis was 32.2 ± 19.1 months in group A and 32.3 ± 22 months in group B. There was no significant difference in the time from onset to diagnosis between group A and B. Motor symptoms were more common than autonomic symptoms in both groups. There was no significant difference in the proportion of initial symptoms between groups A and B. The mean follow-up periods were 52.3 ± 38.3 months in group A and 54.5 ± 40.1 months in group B. There was no significant difference in the follow-up periods. The patients in a bedridden state were most represented in both groups A and B (31% and 37%, respectively), and we found no significant difference in ADL between groups A and B.

Regarding the autonomic symptoms, neurogenic bladder was most highly represented in both groups A and B (90% and 84%, respectively). Among neurogenic bladder, urinary incontinence was most predominant in both groups A and B (52% and 51%, respectively). There was no significant difference in occurrence rate of each autonomic symptom between groups A and B.

### 3.2 Imaging findings

Brain MRI was performed on 78 probable MSA patients (56 MSA-C and 22 MSA-P) (Table 2). The mean age at being performed MRI was 62.6 ± 8.5 in patients with MSA-C and 68.4 ± 7.3 in patients with MSA-P. The mean age at being performed MRI is significantly higher in patients with MSA-P (*p* = 0.002). The mean time from onset was 33.4 ± 23.3 months in patients with MSA-C and 35.5 ± 23.3 months in patients with MSA-P. There was no significant difference in the time from onset between MSA-C and MSA-P. Cerebellar atrophy was found in 43 MSA-C patients (77%) and in 11 MSA-P patents (50%). The frequency of cerebellar atrophy was significantly higher in MSA-C than in MSA-P (*p* = 0.03). Although there was no significant difference in the frequency of pontine abnormalities, the frequency of putaminal abnormalities was significantly higher in patients with MSA-P (73%) than in patients with MSA-C (7%) (*p* < 0.001).

**Table 2:** Imaging findings.

	MSA	MSA-C	MSA-P	PD
MRI				
*n*	78	56	22	
Age	64.2 ± 8.5	62.6 ± 8.5 a	68.4 ± 7.3 a	
The time from onset (month)	34.0 ± 23.2	33.4 ± 23.3	35.5 ± 23.3	
Cerebellar atrophy (%)	54(69)	43(77) b	11(50) b	
Pontine abnormality (%)	48(62)	38(68)	10(45)	
Putaminal abnormality (%)	20(26)	4(7) c	16(73) c	
[^123^I]-MIBG myocardial scintigraphy				
*n*	21	11	10	24
Age	66 ± 6.4	64.2 ± 6.2	68 ± 6.3	69.7 ± 7.5
The time from onset (month)	25.7 ± 24.2	26.6 ± 28.7	24.4 ± 18.8	33.0 ± 29.5
Early H/M ratio	2.12 ± 0.34	2.26 ± 0.36^d^	1.95 ± 0.22 d	1.63 ± 0.31 d
Delayed H/M ratio	2.2 ± 0.40	2.31 ± 0.40 e	2.08 ± 0.40 e	1.48 ± 0.39 e
DaT SPECT				
*n*	8	2	6	17
Age	66.5 ± 8.2	55.5 ± 2.1	70.1 ± 5.4	68.4 ± 9.4
The time from onset (month)	32.0 ± 23.4	40.5 ± 3.5	29.2 ± 27.0	42.4 ± 30.4
Mean SBR	2.27 ± 1.18	2.62 ± 2.6	2.15 ± 0.77	2.47 ± 1.08
Less affected SBR	2.55 ± 1.21	2.77 ± 2.4	2.48 ± 0.94	2.67 ± 1.16
Predominantly affected SBR	1.95 ± 1.2	2.48 ± 2.7	1.78 ± 0.60	2.27 ± 1.03
Asymmetry index (%)	29.5 ± 24.6	32.1 ± 43.1	28.6 ± 21.7	15.1 ± 10.2

MSA, multiple system atrophy; MSA-C, multiple system atrophy with predominant cerebellar ataxia; MSA-P, multiple system atrophy with predominant parkinsonism; PD, Parkinson's disease; H/M ratio, heart/mediastinum ratio; SBR, specific binding ratio.

‘Less affected side’ means the striatum side that showed lower SBR and ‘predominantly side’ means the opposite side.

a *p* = 0.002 by Mann - Whitney *U* test.

b *p* = 0.03 by Fisher's exact test.

c *p* < 0.001 by Fisher's exact test.

d *p* < 0.001 by Kruskal-Wallis test. PD vs MSA-C was *p* < 0.001 and PD vs MSA-P was *p* = 0.021 by the method of Holm.

e *p* < 0.001 by Kruskal-Wallis test. PD vs MSA-C was *p* < 0.001 and PD vs MSA-P was *p* = 0.0018 by the method of Holm.

[^123^I]-MIBG myocardial scintigraphy was performed only on 21 patients in group B. The mean time from onset was 26.6 ± 28.7 months in patients with MSA-C and 24.4 ± 18.8 months in patients with MSA-P. There was no significant difference in time from onset between the patients with MSA-C and MSA-P. The mean early H/M ratio was 2.12 ± 0.34, and the mean delay H/M ratio was 2.2 ± 0.4 in all patients grouped together. In the patients with MSA-C, the mean early H/M ratio was 2.26 ± 0.36, and the mean delay H/M ratio was 2.31 ± 0.4. In the patients with MSA-P, the mean early H/M ratio was 1.95 ± 0.22, and the mean delay H/M ratio was 2.08 ± 0.4. Although not statistically significant, the early H/M ratio tended to be lower in patients with MSA-P than in patients with MSA-C. The mean early and delayed H/M ratio in PD patients was 1.63 ± 0.31 and 1.48 ± 0.39, respectively. The early H/M ratio was significantly lower in patients with PD than in patients with MSA-C and MSA-P (*p* < 0.001 and *p* = 0.021, respectively), and the delayed H/M ratio was significantly lower in patients with PD than in patients with MSA-C and MSA-P (*p* < 0.001 and *p* = 0.0018, respectively).

DAT SPECT was performed in 8 MSA patients (2 MSA-C and 6 MSA-P). The mean time from onset was 40.5 ± 3.5 months in patients with MSA-C and 29.2 ± 27.0 months in patients with MSA-P. There was no significant difference in the time from onset between the patients with MSA-C and MSA-P. Mean SBR/less affected SBR/predominantly affected SBR were 2.62 ± 2.6/2.77 ± 2.4/2.48 ± 2.7 in the MSA-C patients, 2.15 ± 0.77/2.48 ± 0.94/1.78 ± 0.60 in the MSA-P patients and 2.47 ± 1.08/2.67 ± 1.16/2.27 ± 1.03 in the PD patients, respectively. There was no significant difference in mean SBR, less affected SBR and predominantly affected SBR. The AI (%) was 32.1 ± 43.1 in the MSA-C patients, 28.6 ± 21.7 in the MSA-P patients, and 15.1 + 10.2 in the PD patients. There was also no significant difference in AI.

## 4 Discussion

The results of the current study show that the proportion of MSA-P was larger than previously recorded. The following points may be the cause. [^123^I]-MIBG myocardial scintigraphy likely contributes to the differential diagnosis between MSA-P and PD, as [^123^I]-MIBG myocardial scintigraphy has previously been noted as useful for the differential diagnosis between MSA and PD [[5], [6], [7]]. Our results also indicated that MSA patients showed higher early and delayed H/M ratios than did PD patients. However, according to previous reports, some MSA patients showed low H/M ratios [14,15], which agreed with the results in some of our cases with MSA. Additionally, other previous reports showed that the H/M ratios in patients with MSA-P were significantly lower than in patients with MSA-C [16,17], however the results in this study did not corroborate those findings. Thus, the interpretation of [^123^I]-MIBG myocardial scintigraphy in patients with MSA requires careful consideration. Furthermore, the aging of the population could affect the incidence of MSA-P. The increase of age at onset in group B presumably reflected the aging of the population in Japan. Moreover, according to previous reports, the age of the patients at onset of MSA-P was significantly higher than the age of the patients at onset of MSA-C, and the proportion of parkinsonism-onset increased with aging [18,19]. Therefore, there is a possibility that the aging of the population increases the frequency of MSA-P, and we presume that the incidence of MSA-P in Japan will continue to rise in the future.

Although various imaging methods (for example, MRI, [^123^I]-MIBG myocardial scintigraphy and DAT SPECT) are now available, the time from onset to diagnosis was not shortened. We considered that this was because the early stage of MSA sometimes presented with only mono system disturbances, and a certain period was needed to meet the second consensus criteria of MSA. Interestingly, some of the patients in this study were treated for PD in other hospitals, and were then referred to our institution because they displayed an atypical clinical course or a poor response to L-dopa.

There are some limitations to this study. Firstly, the patients in this study were only clinically diagnosed as having MSA, and a pathological examination was not performed. Therefore, there is a possibility that several MSA patients in this study potentially had another neurodegenerative parkinsonism. In 2007, one study [20] classified the pathological features of Japanese MSA patients into 4 types (OPCA = SND type, OPCA-type, SND-type, and MSA-type) using two grading scales [21,22] and reported that the proportion of the OPCA-type of MSA was larger than the SND-type of MSA as a clinical diagnosis. As there is a lack of recent studies focusing on changes in the pathological features of MSA patients in Japan, it is necessary to confirm the recent findings on this subject. Secondly, the analysis of several imaging examinations was insufficient in this study. In particular, standardization of the value in [^123^I]-MIBG myocardial scintigraphy should be conducted to compare each patient. Moreover, this retrospective study targeted only the patients in one institution, which primarily treated referral patients. We presume that typical MSA-C tends to be easier to diagnose than MSA-P and may not need to be referred to our institution for diagnosis. Therefore, there was likely a facility bias in this study, and the proportion of patients with MSA-C could be underestimated. Further prospective multicenter studies with greater numbers of MSA patients should be undertaken in the future.

In conclusion, the proportion of patients with MSA-P in Japan may currently be larger than previously measured and will likely continue to increase in the coming years. The differential diagnosis of parkinsonism should be performed more carefully to ensure that patients with MSA-P are not overlooked.

## Funding

This research did not receive any specific grant from funding agencies in the public, commercial, or not-for-profit sectors.

## Contributions

Yusuke Tokuhara analysed and interpreted data, and wrote the manuscript. Shohei Watanabe designed the study, collected data and assisted in the preparation of the manuscript. Hiroo Yoshikawa made the research plan. All authors critically reviewed the manuscript, approved the final version of the manuscript and agree to be accountable for all aspects of the work in ensuring that questions related to the accuracy or integrity of any part of the work are appropriately investigated and resolved.

## Declaration of competing interest

The authors have no conflicts of interest to declare.
